# Supplementary material for: Anti-Inflammatory Properties of Chemical Probes in Human Whole Blood: Focus on Prostaglandin E2 Production
Source: Front Pharmacol. 2020 May 6;11:613. doi: 10.3389/fphar.2020.00613 (PMC7218097; doi:10.3389/fphar.2020.00613)
Supplement: Supplementary file 1 [file Table_1.docx]

| **ID** | **Diagnosis** | **Sex** | **Age** | **Treatment** | **Induced PGE_2_** (ng/mL) | **Induced TXB_2_** (ng/mL) | **Induced IL-8** (pg/mL) |
| --- | --- | --- | --- | --- | --- | --- | --- |
| HC018 | Healthy donor | Male | 27 | n/a | 53.3 | 15.5 | Not tested |
| HC019 | Healthy donor | Female | 27 | n/a | 13.4 | 9.5 | Not tested |
| HC27 | Healthy donor | Female | 44 | n/a | 55.4 | 30.1 | 160 |
| HC28 | Healthy donor | Female | 40 | n/a | 48.2 | 23.5 | 360 |
| ULTRA030 | SLE | Male | 38 | Prednisolone, CellCept® | 30.7 | 11.8 | Not tested |
| ULTRA031 | DM | Male | 45 | Prednisolone, MTX | 9.0 | 6.5 | 120 |
| ULTRA032 | DM | Male | 54 | Prednisolone, MTX, Orencia® | 23.6 | 13.7 | 300 |
| ULTRA033 | SLE | Male | 81 | Prednisolone, MabThera®, Plaquenil® | 6.2 | 4.1 | 170 |
| ULTRA034 | SLE | Female | 61 | Plaquenil® | 31.4 | 18.2 | 220 |
| ULTRA035 | SLE | Female | 51 | Prednisolone, Plaquenil® | 132.8 | 32.6 | 1050 |
| ULTRA036 | DM | Male | 63 | No treatment | 96.6 | 12.5 | 230 |
| ULTRA037 | SLE | Female | 59 | Medrol®, Plaquenil®, Trombyl® | 5.0 | 2.6 | 160 |
| ULTRA038 | SLE | Female | 49 | No treatment | 45.3 | 12.5 | 170 |
| ULTRA039 | SLE | Female | 46 | MTX, Plaquenil® | 40.5 | 87.6 | 270 |
| ULTRA040 | SLE | Female | 75 | MTX, Plaquenil® | 5.7 | 3.8 | 490 |
| ULTRA041 | SLE | Female | 33 | Prednisolone, CellCept®, Plaquenil® | 26.4 | 8.6 | 190 |

**Supplementary Table 1.** Patient and healthy donor description. DM; dermatomyositis; MTX, methotrexate; SLE, systemic lupus erythematosus.
